# Differences in lung and lobe volumes between supine and upright computed tomography in patients with idiopathic lung fibrosis

**DOI:** 10.1038/s41598-022-24157-x

**Published:** 2022-11-12

**Authors:** Shotaro Chubachi, Satoshi Okamori, Yoshitake Yamada, Minoru Yamada, Yoichi Yokoyama, Yuki Niijima, Hirofumi Kamata, Makoto Ishii, Koichi Fukunaga, Masahiro Jinzaki

**Affiliations:** 1 Division of Pulmonary Medicine, Department of Medicine, Keio University School of Medicine, 35 Shinanomachi, Shinjuku-ku, Tokyo, 160-8582 Japan; 2 Department of Radiology, Keio University School of Medicine, 35 Shinanomachi, Shinjuku-ku, Tokyo, 160-8582 Japan; 3 Office of Radiation Technology, Keio University Hospital, 35 Shinanomachi, Shinjuku-ku, Tokyo, 160-8582 Japan

**Keywords:** Respiratory distress syndrome, Respiration

## Abstract

No clinical study has compared lung or lobe volumes on computed tomography (CT) between the supine and standing positions in patients with idiopathic lung fibrosis (IPF). This study aimed to compare lung and lobe volumes between the supine and standing positions and evaluate the correlations between the supine/standing lung volumes on CT and pulmonary function in patients with IPF. Twenty-three patients with IPF underwent a pulmonary function test and both low-dose conventional (supine position) and upright CT (standing position) during inspiration breath-holds. The volumes of the total lungs and lobes were larger in the standing than in the supine position in patients with IPF (all p < 0.05). Spearman's correlation coefficients between total lung volumes on chest CT in supine/standing positions and vital capacity (VC) or forced VC (FVC) were 0.61/0.79 or 0.64/0.80, respectively. CT-based volumes on upright CT were better correlated with VC and FVC than those on supine CT. Lung and lobe volumes in the standing position may be useful biomarkers to assess disease severity or therapeutic effect in patients with IPF.

## Introduction

Idiopathic pulmonary fibrosis (IPF) is a debilitating and fatal scarring lung disease^1^. It is the most common interstitial lung disease (ILD)^2,3^ and is characterized by chronic, progressive, fibrosing interstitial pneumonia of unknown cause with an usual interstitial pneumonia (UIP) pattern^4^. Pulmonary function tests (PFTs) reflect the histologic severity of the disease better than symptoms or chest radiography^5^. It is the most standardised approach for objectively monitoring and quantifying disease progression. However, it requires additional time and effort to obtain, has variable reproducibility^6^ and cannot be performed adequately by some patients who are unable to cooperate with forced inhalation or exhalation^7^.

Chest computed tomography (CT) has become the standard of care for the diagnostic evaluation of patients with IPF^4^. Many quantitative methods of computer-aided analysis of chest CT^8–11^ have been reported for IPF to quantify severity and to monitor disease progression. These quantitative analyses of chest CT may be an alternative to pulmonary function for the evaluation of IPF severity^8,9^. The correlation between lung volumes on CT and pulmonary function has been evaluated in healthy volunteers^12^ and patients with chronic obstructive pulmonary disease^13,14^. However, very few reports have evaluated the association between lung volume on CT and PFTs in patients with IPF^15,16^. In addition, there are no reports on the correlation between regional (e.g. lobar) volume on chest CT and pulmonary function in patients with IPF.

Recently, a 320-detector-row upright CT scanner was developed to evaluate human anatomy in the standing position three-dimensionally and to clarify the effects of gravity on the entire human body^17^. In a previous report of healthy volunteers, we reported that the bilateral lung volumes were significantly higher in the standing position than in the supine position using upright and supine CT scanners^12,18^. To the best of our knowledge, no clinical studies to date have accurately compared lung volumes of patients with IPF in the supine and standing positions. We hypothesised that the lung volumes between the supine and standing positions would be different and that compared with supine CT, upright CT-based volumes would be more correlated with PFT measurements in patients with IPF. The aims of this study were as follows: (1) to compare the lung and lobe volumes between the supine and standing positions, and (2) to compare the correlations between the supine/standing lung and lobe volumes on CT and pulmonary function in patients with IPF.

## Methods

### Study population

**Figure 1:**
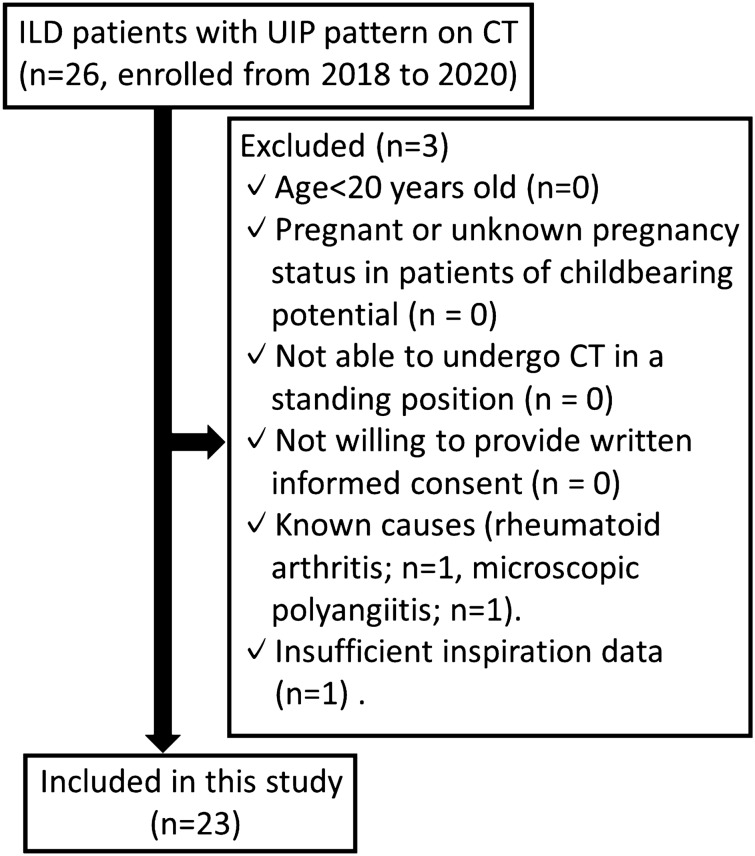
Flowchart of participant inclusion and exclusion. *ILD* interstitial lung disease, *UIP* usual interstitial pneumonia, *CT* computed tomography.

This prospective study was approved by the institutional review board of Keio University School of Medicine. Written informed consent was obtained from all patients [UMIN Clinical Trials Registry (UMIN-CTR): UMIN000026587]. All methods were carried out in accordance with the relevant guidelines and regulations. From October 2018 to September 2020, a total of 26 consecutive patients with known ILD and with radiological diagnosis of UIP pattern, who were scheduled for clinical CT examination, were considered for this prospective study. To determine the diagnostic categories of CT features according to the guidelines of the American Thoracic Society/European Respiratory Society/Japanese Respiratory Society/Latin American Thoracic Association 2018^4^, each CT scan was evaluated independently by a pulmonologist with 10 years of experience (S.O.) and a chest radiologist with 15 years of experience (Y. Y.) who were blinded to all clinical information. If the CT scans were discordantly scored between readers 1 and 2, the CT features were determined by mutual agreement. The exclusion criteria were as follows: aged < 20 years, pregnant or unknown pregnancy status in patients of childbearing potential (n = 0); inability to undergo CT in a standing position (n = 0); lack of willingness to provide written informed consent (n = 0); known causes (rheumatoid arthritis, n = 1; microscopic polyangiitis, n = 1) and insufficient inspiration data (n = 1). Finally, a total of 23 patients were included in this study (Fig. 1).

### CT imaging protocol

All patients underwent both conventional chest low-dose CT in the supine position with arms raised using a 320-detector-row CT (Aquilion ONE, Canon Medical Systems, Otawara, Japan) and upright chest low-dose CT in a standing position with arms down performed using a 320-detector-row upright CT (prototype TSX-401R; Canon Medical Systems) in a randomised order within 1 h on the same day^12,17–19^. These chest CT scans in the two positions were unenhanced and were performed during deep inspiration breath-hold with automatic exposure control using a noise index of 24 HU for a slice thickness of 5 mm (tube current range, 10–350 mA)^12,18,19^. Other scanning parameters were also the same for supine and standing chest CT scans: peak tube voltage, 120 kVp; rotation speed, 0.5 s; slice collimation, 0.5 mm × 80; field of view, 400 mm; and pitch factor, 0.813. The series of contiguous 0.5-mm-thick images was reconstructed using Adaptive Iterative Dose Reduction 3D (Canon Medical Systems)^12,18–20^.

### PFT

The PFT was performed in a stable condition, with the patient in a sitting position, using a spirometer (Chestac-8900, Chest M.I., Tokyo, Japan) in accordance with ATS/European Respiratory Society recommendations^12,21,22^. The predicted values of spirometric measurements were derived from the guidelines of the Japanese Respiratory Society^12,19,23^.

### Lung and lobe volume measurements using CT

**Figure 2:**
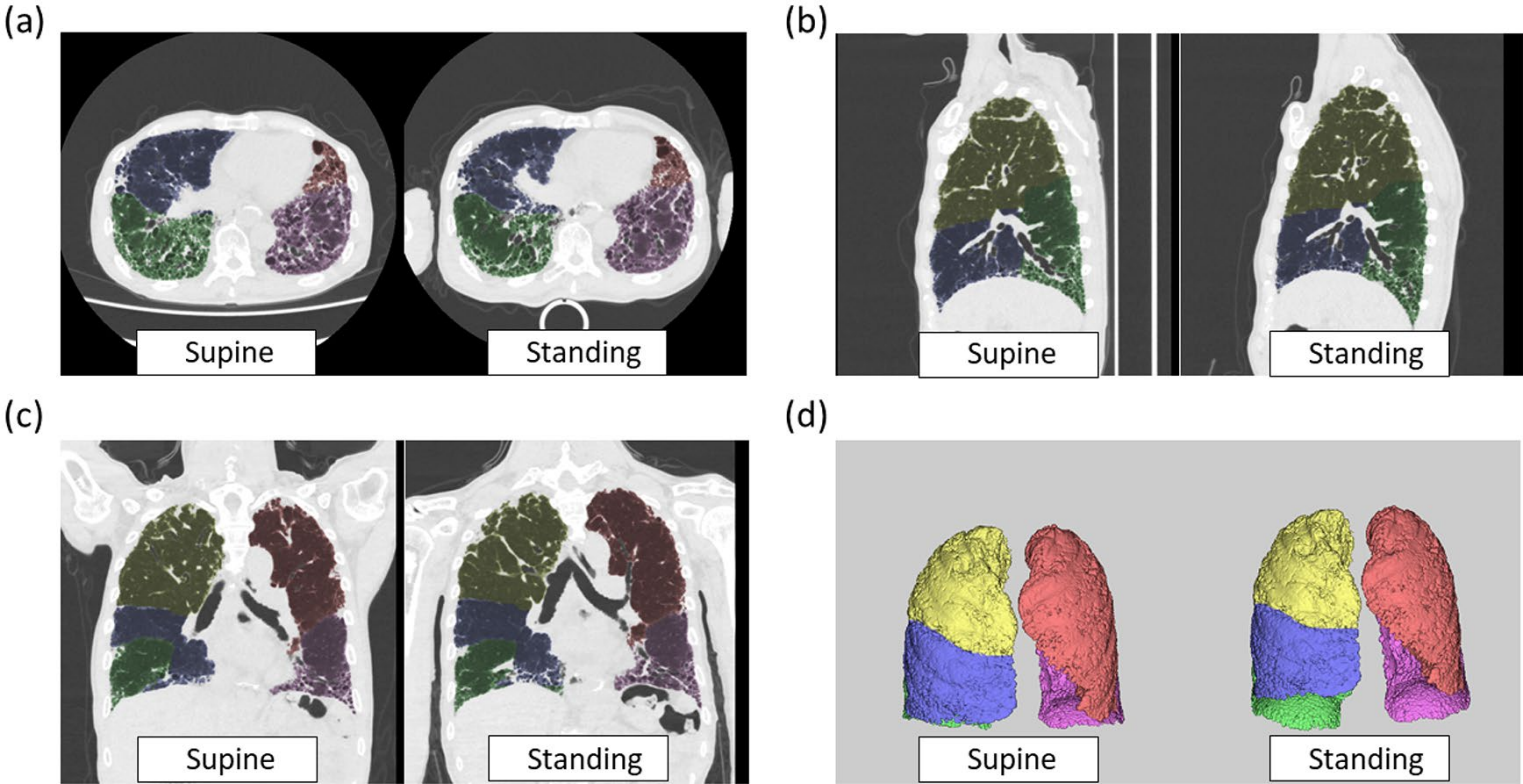
Representative lung and lobe volume measurements in a 71-year-old male patient with IPF. Axial images (**a**), sagittal images (**b**), coronal images (**c**), and volume rendering lung/lobe images (**d**) acquired in the supine and standing positions. Yellow indicates the right upper lobe, blue is the right middle lobe, green is the right lower lobe, pink is the left upper lobe, and purple is the left lower lobe. *IPF* idiopathic pulmonary fibrosis.

Lung and lobe volume measurements on CT for all 24 patients in each position were performed by a pulmonologist with 11 years of experience (S.C.) using a commercially available workstation (Synapse Vincent; Fuji Film Co., Ltd., Tokyo, Japan)^12,18,19,24–26^. This workstation incorporated a lobar computer-aided diagnosis (CAD) system that was previously demonstrated to precisely measure lobar volumes^12,18,27^. This system automatically extracted the right and left lungs, recognised the lobar bronchi, and determined the locations of the fissures (Fig. 2)^12,18,28^. The pulmonologist verified the results of segmentation by CAD and made manual corrections by delineating fissures when the CAD system failed to identify fissures properly, as described in a previous study^12,18,28^. During all measurements, the pulmonologist was blinded to the participants’ characteristics and the results of the PFT. The ratios of the volume of the total (bilateral) lung, each lung, and each lobe in the standing position to those in the supine position were then calculated^12,18^. The proportional volumes of each lung and each lobe relative to the total lung volume were also calculated^12,18^.

### CT fibrosis score

Pulmonologist with 11 years of experience (S.C.) scored ground glass opacity (CT-alveolar score) and reticular opacity (CT-interstitial score) on a scale of 0–5, as previously reported^29^. These scores were also summed into a total CT score (CT-total score)^29^.

### Statistical analysis

Data are presented as medians [interquartile range (IQR)]. The Wilcoxon signed-rank test was performed to analyse the differences in the volumes of the total lung, right lung, left lung, and each lobe between the supine and standing positions; differences in the ratios of volumes in the standing position to those in the supine position among lobes; and differences in the proportional volumes of each lung and each lobe relative to the total lung volume between the supine and standing positions^12,19^. Bonferroni correction was used for multiple comparisons^12,19^. The association between the volumes on CT in each position and parameters on PFT, and the association between CT fibrosis score and ratio of volume in the standing position to that in the supine position were evaluated using Spearman’s correlation test^19^. The significance level for all tests was 5% (two-sided). All data were analysed using a commercially available software program (JMP version 14; SAS Institute Inc., Cary, NC, USA).

### Ethics approval and consent to participate

This study was registered in the University Hospital Medical Information Network (UMIN 000026587) and was approved by the ethics committees of Keio University and its affiliated hospitals (No. 20160385). All methods were carried out in accordance with all relevant guidelines and regulations.

## Results

### Clinical features of the study population

Table 1 presents the baseline characteristics of the study population. The median age of the patients with IPF was 76 years (IQR, 72–81), of which 30.4% were women. The median VC, % predicted and FVC, % predict were 72.5 and 72.8%, respectively (IQR, 54.0–81.2 and 56.7–83.4, respectively).

**Table 1:** Clinical features of the study population.

N	23
Sex, female, (%)	7 (30.4)
Age, years	76 (72–81)
Smoking index, pack-years	26.0 (0–48.0)
Current smokers, (%)	0 (0)
Height, cm	161.4 (152.2–165.8)
Weight, cm	59.8 (52.7–65.0)
Body mass index	23.8 (21.3–26.6)
**Pulmonary function test**	
VC, ml	1970.0 (1670.0–2610.0)
VC, % predicted	72.5 (54.0–81.2)
FVC, ml	1860.0 (1650.0–2460.0)
FVC, % predicted	72.8 (56.7–83.4)

Data are presented as median (IQR) or number (%).

*VC* vital capacity, *FVC* forced vital capacity.

### Comparison of the lung and lobe volumes on CT between the supine and standing positions

The lung and lobe volumes on CT scans are shown in Table 2 and Supplementary Fig. S1. The bilateral lung, right lung, right upper lobe, right middle lobe, right lower lobe, left lung, left upper lobe, and left lower lobe volumes were significantly higher in the standing position than in the supine position (all p < 0.05). The ratio of the right middle lobe volume in the standing position (1.04; IQR, 0.99–1.09) to that in the supine position was significantly lower than that of the right upper lobe volume (1.09; IQR, 1.04–1.15) (P < 0.0001) and that of the right lower lobe volume (1.11; IQR, 1.06–1.22) (P < 0.0001). The ratio of the left lower lobe volume in the standing position to that in the supine position (1.08; IQR, 1.06–1.23) was significantly greater than that of the left upper lobe volume (1.07; IQR, 1.03–1.11) (p = 0.0005). The correlations between CT fibrosis scores and ratios of volume in the standing position to that in the supine position are shown in Supplementary Fig. S2. In a few lobes, the alveolar score and total score were weakly correlated with the volume ratio. However, interstitial scores were not correlated with it.

**Table 2:** Lung and lobe volumes between the supine and standing positions.

	Lung and lobe volumes on CT, ml		*p* value	Ratio of volume in the standing position to that in the supine position
	Supine	Standing	Supine vs. standing	
Bilateral lungs	2815.4 (2209.2–3086.2)	2887.7 (2363.1–3624.8)	< 0.0001	1.07 (1.05–1.16)
Right lung	1644.4 (1231.6–1801.8)	1752.5 (1328.6–1955.3)	< 0.0001	1.08 (1.03–1.16)
Right upper lobe	650.4 (510.0–771.2)	664.7 (582.4–862.0)	< 0.0001	1.09 (1.04–1.15)^a^
Right middle lobe	377.7 (295.2–475.0)	376.0 (304.8–488.1)	0.0272	1.04 (0.99–1.09)^a^
Right lower lobe	484.5 (369.7–584.2)	558.7 (411.8–674.9)	< 0.0001	1.11 (1.06–1.22)^a^
Left lung	1190.4 (940.6–1462.5)	1342.3 (1026.4–1674.9)	< 0.0001	1.08 (1.05–1.15)
Left upper lobe	720.5 (562.3–916.3)	784.5 (603.3–971.4)	< 0.0001	1.07 (1.03–1.11)^b^
Left lower lobe	462.2 (349.4–567.6)	539.3 (378.0–693.5)	< 0.0001	1.08 (1.06–1.23)^b^

Data are presented as medians (interquartile ranges).

^a^The ratio of the right middle lobe volume in the standing position to that in the supine position was significantly lower than that of the right upper and lower lobe volume (both p < 0.0001).

^b^The ratio of the left lower lobe volume in the standing position to that in the supine position was significantly greater than that in the left upper lobe volume (p = 0.0005).

### Proportional volumes of each lung and each lobe relative to the total lung volume in the supine and standing positions

The proportional volumes of the right middle and left upper lobes relative to the total lung volume were significantly lower in the standing position than in the supine position, whereas those of the bilateral lower lobes were significantly greater in the standing position than in the supine position (all p < 0.01; Table 3 and Supplementary Fig. S3).

**Table 3:** Lung and lobe volumes relative to the total lung volume in supine and standing positions.

	Proportional volumes of each lung and each lobe relative to the total lung volume, %		*p* value
	Supine	Standing	Supine vs. standing
Bilateral lungs	100 (100–100)	100 (100–100)	
Right lung	56.3 (51.0–60.6)	56.3 (50.9–59.1)	0.5155
Right upper lobe	23.5 (20.9–28.3)	23.4 (21.1–26.8)	0.5749
Right middle lobe	14.5 (11.7–17.3)	14.1 (10.8–16.1)	< 0.0001
Right lower lobe	17.6 (14.4–20.2)	18.2 (15.0–19.9)	0.0005
Left lung	43.7 (39.4–49.0)	43.7 (40.9–49.1)	0.5155
Left upper lobe	26.5 (25.3–30.8)	26.0 (25.0–30.2)	0.0028
Left lower lobe	16.6 (14.9–19.0)	17.7 (16.0–19.4)	0.0005

Data are presented as medians (interquartile range).

### Correlations of lung and lobe volumes in the supine and standing positions with the results of the PFT

The correlations of lung and lobe volumes in the supine and standing positions with the results of the PFT are shown in Table 4 and Supplementary Figs. S4 and S5. Volumes of the total lung, right lung, right upper lobe, left lung, and left upper lobe in the supine and standing positions were significantly correlated with VC (total lung: ρ = 0.61 vs. ρ = 0.79; right lung: ρ = 0.48 vs. ρ = 0.73; right upper lobe: ρ = 0.54 vs. ρ = 0.64; left lung: ρ = 0.51 vs. ρ = 0.70; left upper lobe: ρ = 0.62 vs. ρ = 0.65, respectively; all p < 0.05) and FVC (total lung: ρ = 0.64 vs. ρ = 0.80; right lung: ρ = 0.50 vs. ρ = 0.74; right upper lobe: ρ = 0.56 vs. ρ = 0.65; left lung: ρ = 0.54 vs. ρ = 0.71; left upper lobe: ρ = 0.64 vs. ρ = 0.65, respectively; all p < 0.05). Left lower lobe volumes in the supine position were not correlated with VC (ρ = 0.31; p = 0.155) and FVC (ρ = 0.34; p = 0.1136), whereas left lower lobe volumes in the standing position were significantly correlated with VC (ρ = 0.55; p < 0.01) and FVC (ρ = 0.58; p < 0.01).

**Table 4:** Correlations of lung and lobe volumes in the supine and standing positions from the PFT.

	Supine	Standing
**VC**		
Bilateral lungs	0.61**	0.79**
Right lung	0.48*	0.73**
Right upper lobe	0.54**	0.64**
Right middle lobe	0.40	0.40
Right lower lobe	0.16	0.36
Left lung	0.51*	0.70**
Left upper lobe	0.62**	0.65**
Left lower lobe	0.31	0.55**
**FVC**		
Bilateral lungs	0.64**	0.80**
Right lung	0.50**	0.74**
Right upper lobe	0.56**	0.65**
Right middle lobe	0.40	0.38
Right lower lobe	0.18	0.38
Left lung	0.54**	0.71**
Left upper lobe	0.64**	0.65**
Left lower lobe	0.34	0.58**

*PFT* pulmonary function test, *VC* vital capacity, *FVC* forced vital capacity.

* and ** indicate p < 0.05 and p < 0.01, respectively.

## Discussion

Our findings demonstrated differences in lung and lobe volumes between the supine and standing positions, as assessed by chest CT scans in patients with IPF. The volume of the total lungs, bilateral lungs, bilateral upper lobes, right middle lobe, and bilateral lower lobes were significantly greater in the standing position than in the supine position, with lower lobes showing larger changes. These results are consistent with those of our previous study in healthy volunteers^18^. Upright CT may provide more physiologic relevant images and accurate prediction of pulmonary function in IPF patients than the conventional supine CT.

In this study, the total lung volume was smaller than that in our previous study in healthy volunteers^18^. Previous studies have identified molecular and cellular mechanisms that are potentially associated with the onset and progression of IPF^30,31^. The excessive production of extracellular matrix by lung myofibroblasts leads to progressive stiffening of the lung tissue^30^. Recently, Jaffar et al. revealed that fibroblasts from patients with IPF were stiffer than those from donors without IPF^31^. Regions with extensive fibrosis might not undergo volume changes as much as nonfibrotic regions. In this study, a few lobes with high alveolar scores were weakly correlated with the ratio of the volume in the standing position to that in the supine position. However, lobes with extensive fibrosis (high interstitial score) did not show smaller changes than nonfibrotic lobes. The reason for these results might be that the number of patients was small, and that fibrosis was evaluated qualitatively. Evaluation of lung volume using upright CT may be a useful biomarker that reflects the pathogenesis of IPF.

Our study demonstrated that there was significant correlation between total lung volumes in the standing and supine positions, and pulmonary function in patients with IPF. These results were consistent with previous reports using conventional CT with both automated software^15^ and manual measurements^16^. Our study also demonstrated that upright CT could predict VC and FVC more precisely as compared to conventional supine CT. It has been reported that lung volume and flow distribution change heterogeneously across the lung lobes of IPF patients, with reduced capacity in the lower lobes^32^. However, there are no reports on the correlation between regional (e.g., lobar) volume on chest CT and pulmonary function in patients with IPF. In this study, lower lobe volumes correlated more strongly with lung function in the upright CT than in the conventional CT. The difference of the lobe volumes ratio in the standing and the supine positions might be the cause for this difference. The volume of lower lobes changes more dramatically during breathing than that of the upper lobes because of the effects of gravity on lung recoil^33–35^. Also, this difference might be caused by the change in the diaphragm movement between the two positions. The diaphragm settles lower in the standing position, allowing for greater expansion of the lower lobes than in the supine position. A previous report showed that diaphragmatic mobility is lower in IPF patients than in healthy controls^36^. In this study, the lower lobe volumes in the standing position, and the changes in the volumes between the supine and standing positions were smaller in patients with IPF than in the healthy participants of our previous study^12,18^. Thus, clinicians may use the volumes of the lower lobes on upright CT as a new clinical indicator to assess disease severity or therapeutic effect in IPF patients. Specifically, these indicators could be used as an alternative tool to predict disease severity and disease course of IPF in situations in which a PFT cannot be performed, such as in elderly patients.

The approval of medical treatments for IPF marks a new era in approaching this deadly disease, offering hope to patients and their physicians, a clearer path forward for companies interested in the development of new treatments, and the potential for new biological insights^37^. Nintedanib^38^ and pirfenidone^39^ are promising drugs that suppress the progression of IPF. To measure treatment outcomes, changes in FVC have been frequently used^38,39^. However, it requires additional time and effort to obtain, has variable reproducibility^6^ and cannot be performed adequately by some patients. Moreover, to reduce the spread of severe acute respiratory syndrome coronavirus 2, many pulmonary function testing laboratories have significantly reduced their testing capacity^40^. One of the strengths of the present results is that upright CT could predict FVC more precisely than conventional supine CT. Recently, few studies have shown that changes in fibrosis quantitatively using a computer-aided system were correlated with changes in pulmonary function under treatment^41,42^. In the future, we need to evaluate whether quantitative analysis of lung volume using upright CT is useful for treatment outcomes in IPF patients.

The first investigations of chest CT in ILD during the late 1980s and the early 1990s marked a golden era of CT-pathological correlative studies^43^. Recently, chest CT data have been integrated with pulmonary function in staging models to predict prognosis in patients with IPF^44^. In contrast to pulmonary emphysema, the patterns of ILD are quite heterogeneous in morphologic characteristics and lack a standard density threshold that can dichotomise the visualised lung tissue into normal and diseased tissues^45^. Nevertheless, the global histogram of density metrics of CT images—skewness, kurtosis, and mean lung density—are helpful in estimating the extent of ILD^8^ and prognosis of patients with IPF^9^. However, the relationship between lung volume evaluated by chest CT and prognosis in patients with IPF has not been investigated. Future studies investigating the relationship between lung volumes using upright CT and the prognosis of patients with IPF are needed.

This study had several limitations. First, this was a prospective study with a small sample size and was conducted at a single institution. Second, the percentage of patients with severe IPF was relatively low. Further studies with larger sample sizes and a representative percentage of patients with severe IPF are needed. Third, in this study, upright CT was performed with arms lowered, whereas conventional supine CT was performed with arms raised; thus, the form of the chest would have been slightly different between upright and supine positions, which may have influenced the results of this study. However, we believe that standing with the arms lowered is the natural standing posture for humans. Fourth, we could not repeat CT scans and PFT measurements. Thus, we could not evaluate the reproducibility of our results.

## Conclusion

Upright and supine CT quantification of the lung and lobe volumes in patients with IPF revealed differences in the lung and lobe volumes between the supine and standing positions, and that the lung and lobe volumes in the standing position are useful biomarkers for predicting VC and FVC. Evaluation of lung and lobe volumes using upright CT should be incorporated into future IPF studies. CT-based volumes on upright CT were better correlated with VC and FVC determined by PFT than those on supine CT in patients with IPF. Lung and lobe volumes in the standing position may be useful biomarkers to assess disease severity or therapeutic effects in patients with IPF.

## Supplementary Information

Supplementary Information.

## Data Availability

The datasets generated and/or analysed during the current study are available from the corresponding author upon reasonable request.

## Abbreviations

IPF: Idiopathic lung fibrosis
ILD: Interstitial lung disease
UIP: Usual interstitial pneumonia
CT: Computed tomography
PFT: Pulmonary function test
VC: Vital capacity
FVC: Forced vital capacity
