# Supplementary material for: Differences in lung and lobe volumes between supine and upright computed tomography in patients with idiopathic lung fibrosis
Source: Sci Rep. 2022 Nov 12;12:19408. doi: 10.1038/s41598-022-24157-x (PMC9653373; doi:10.1038/s41598-022-24157-x)
Supplement: Supplementary file 1 — Supplementary Information. [file 41598_2022_24157_MOESM1_ESM.docx]

# Supplementary material

**Differences in Lung and Lobe Volumes Between Supine and Upright Computed Tomography in Patients with Idiopathic Lung Fibrosis**

Shotaro Chubachi, Satoshi Okamori, Yoshitake Yamada, Minoru Yamada, Yoichi Yokoyama, Yuki Niijima, Hirofumi Kamata, Makoto Ishii, Koichi Fukunaga & Masahiro Jinzaki

**
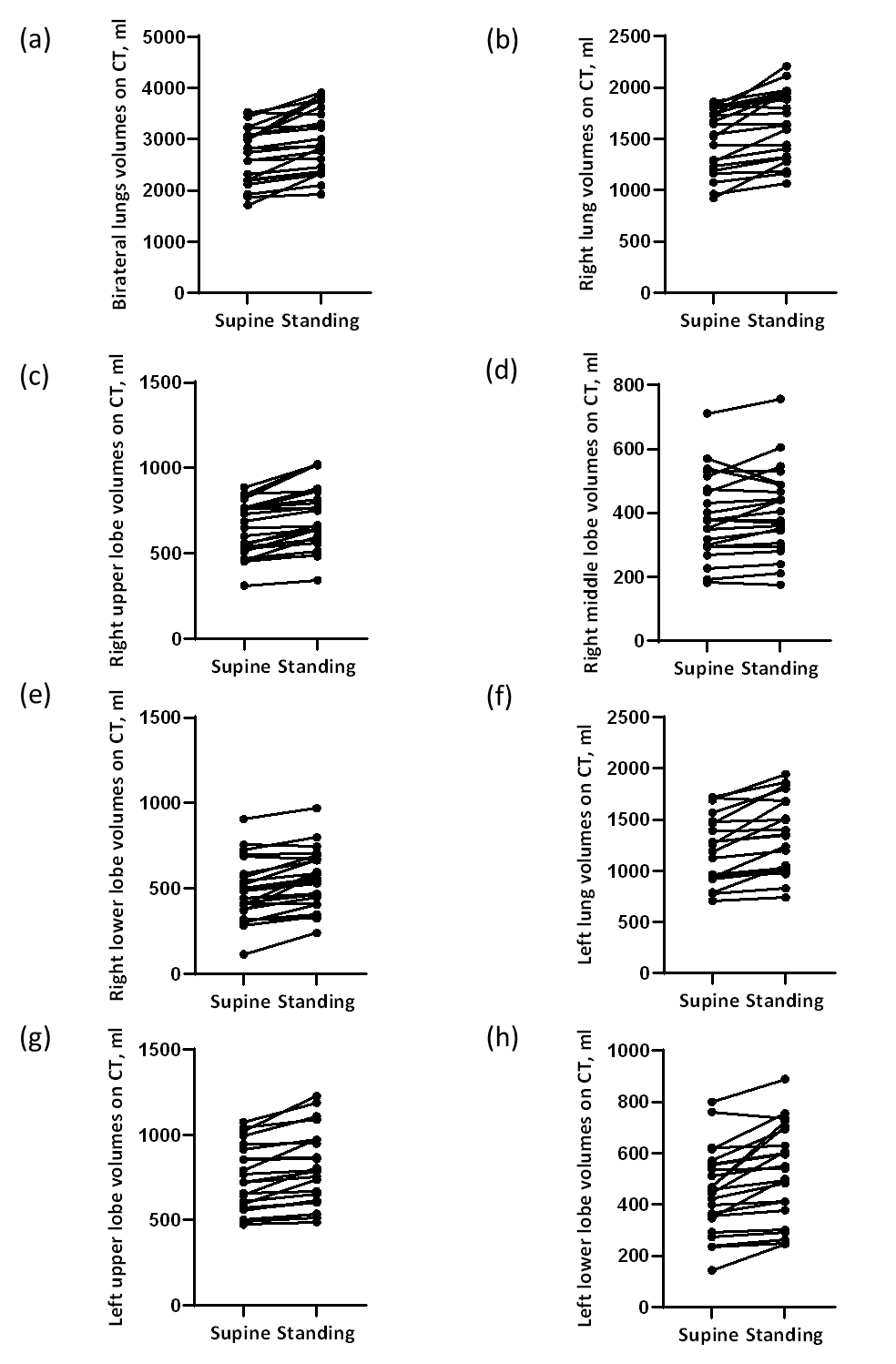
**

**Supplementary Figure 1.** Differences in lung and lobe volumes between the supine and standing positions. Differences in (**a**) bilateral lung, (**b**) right lung, (**c**) right upper lobe, (**d**) right middle lobe, (**e**) right lower lobe, (**f**) left lung, (**g**) left upper lobe, and (**h**) left lower lobe volumes on CT between the supine and standing positions. CT, computed tomography.

**
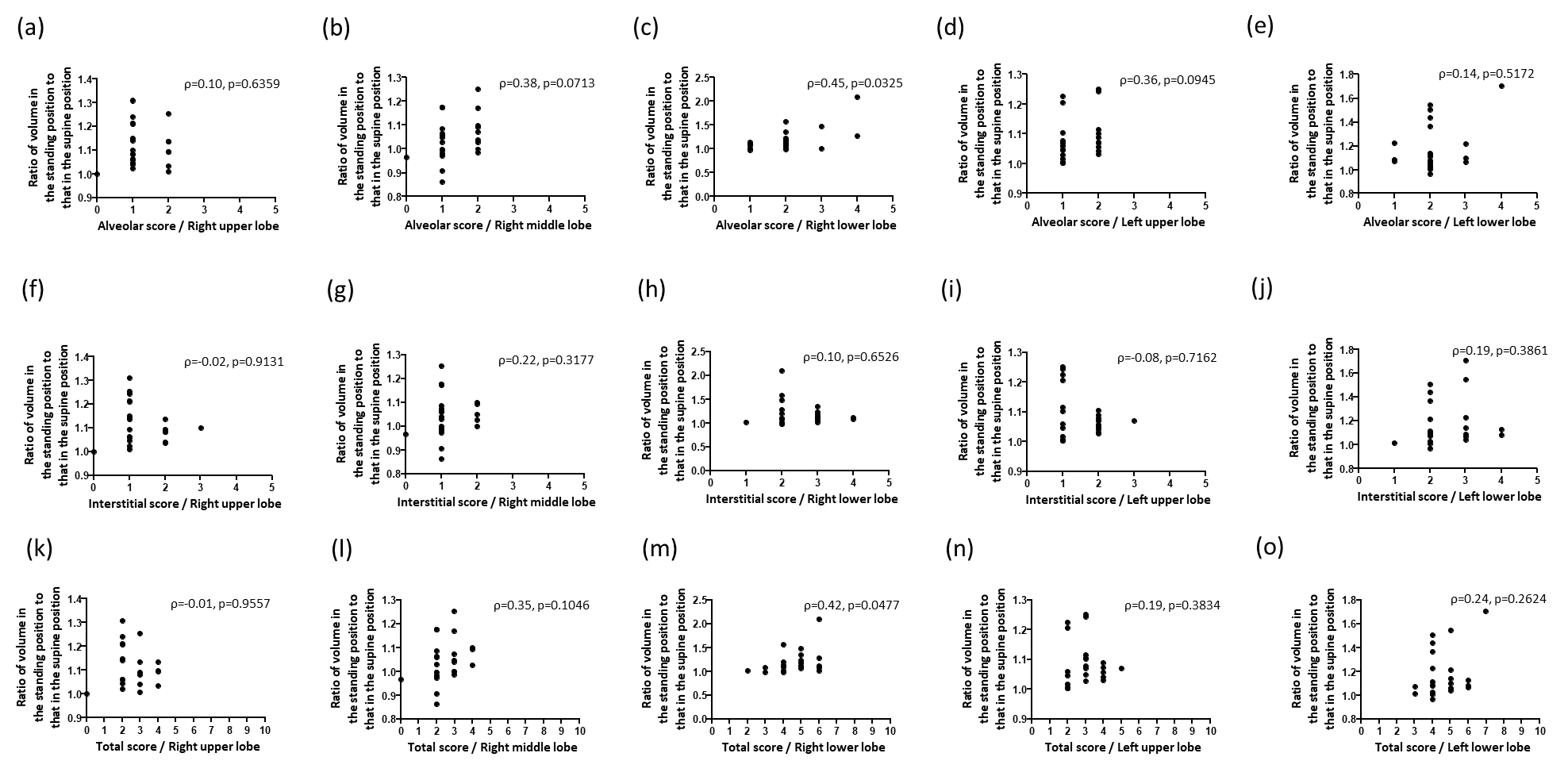
**

**Supplementary Figure 2.** Correlation of CT fibrosis scores with the ratios of the volume in the standing position to that in the supine position. Correlation of the CT alveolar score with the ratio of the volume in the standing position to that in the supine position for the (**a**) right upper lobe, (**b**) right middle lobe, (**c**) right lower lobe, (**d**) left upper lobe, and (**e**) left lower lobe. Correlation of the CT interstitial score and the ratio of the volume in the standing position to that in the supine position for the (**f**) right upper lobe, (**g**) right middle lobe, (**h**) right lower lobe, (**i**) left upper lobe, and (**j**) left lower lobe. Correlation of the CT total score and the ratio of the volume in the standing position to that in the supine position for the (**k**) right upper lobe, (**l**) right middle lobe, (**m**) right lower lobe, (**n**) left upper lobe, and (**o**) left lower lobe. CT, computed tomography.

**
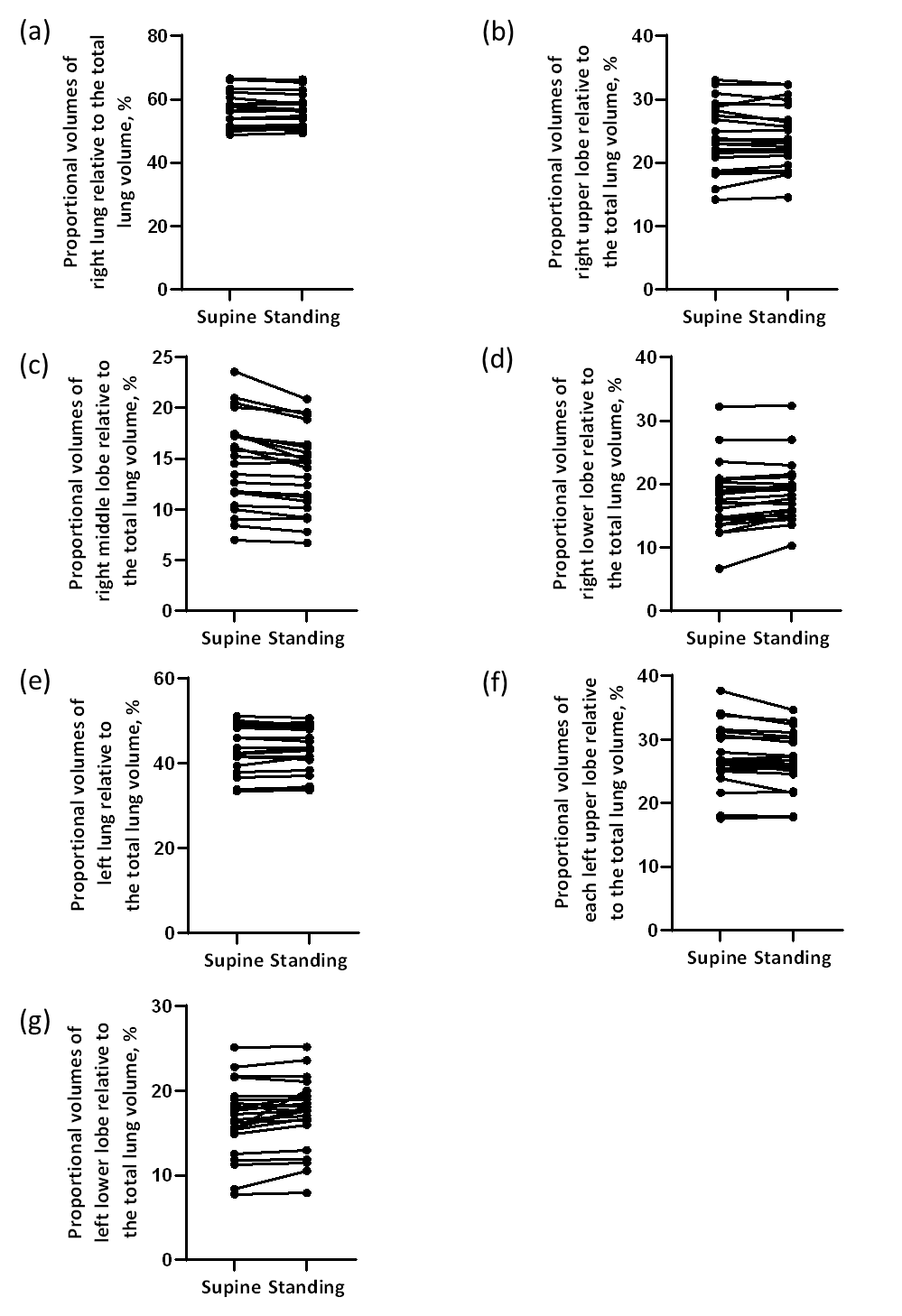
**

**Supplementary Figure 3.** Lung and lobe volumes relative to the total lung volumes in the supine and standing positions. Proportional volumes (in %) of the (**a**) right lung, (**b**) right upper lobe, (**c**) right middle lobe, (**d**) right lower lobe, (**e**) left lung, (**f**) left upper lobe, and (**g**) left lower lobe relative to the total lung volume.

**
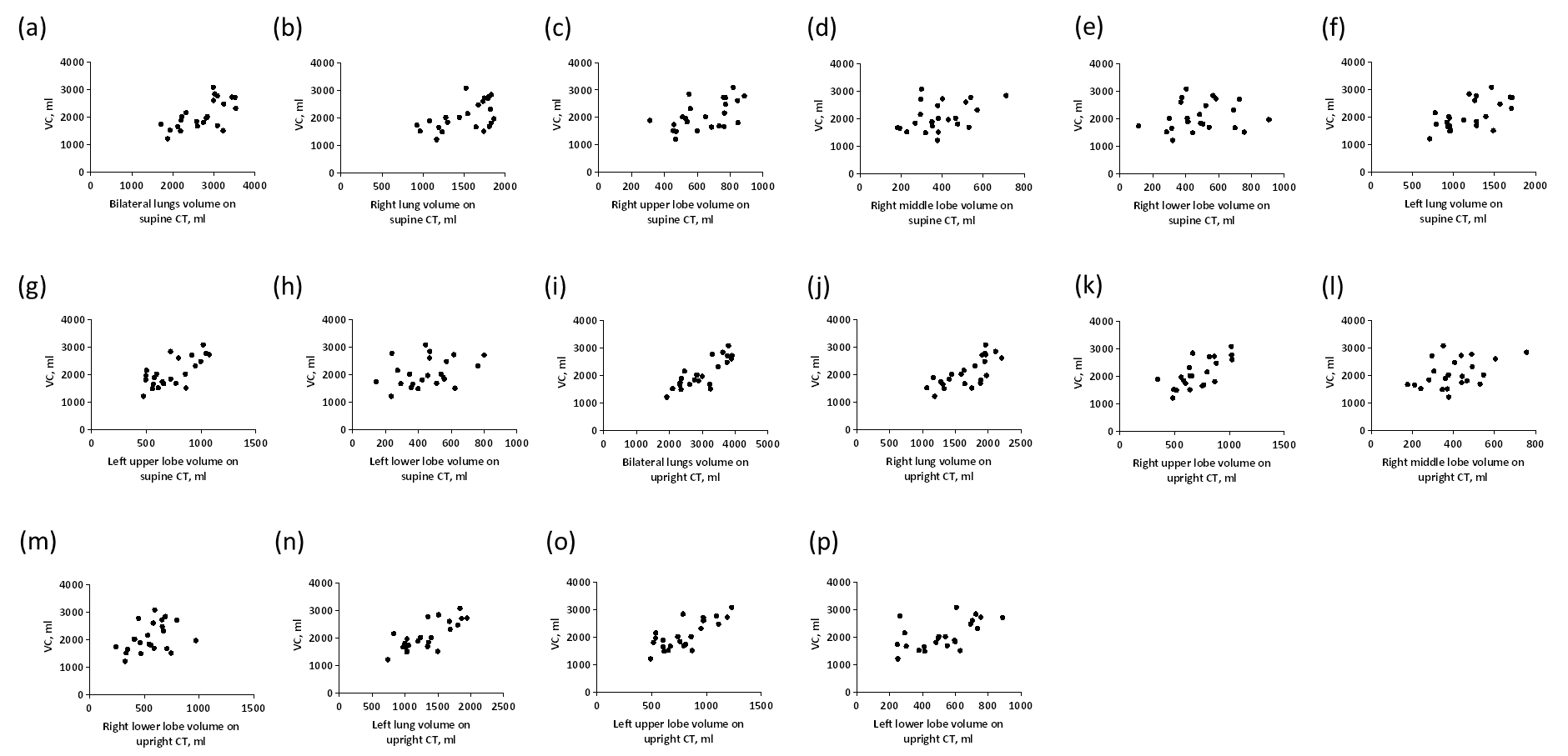
**

**Supplementary Figure 4.** Correlations of lung and lobe volumes in the supine and standing positions with VC based on the PFT results. PFT, pulmonary function test; VC, vital capacity.

**
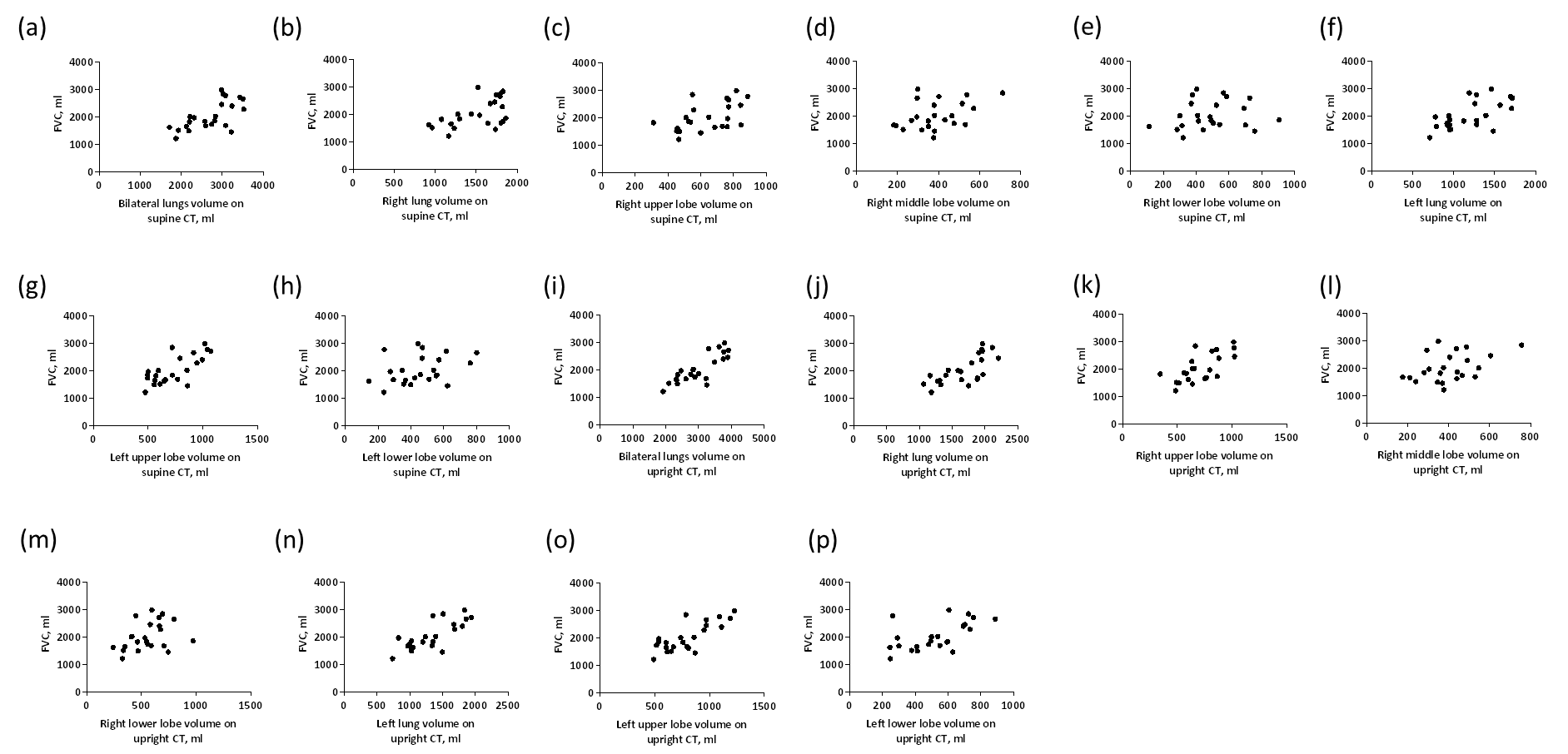
**

**Supplementary Figure 5.** Correlations of lung and lobe volumes in the supine and standing positions with FVC based on the PFT results. PFT, pulmonary function test; FVC, forced vital capacity.
